# Hydrogen-Rich Gas Production by Cogasification of Coal and Biomass in an Intermittent Fluidized Bed

**DOI:** 10.1155/2013/276823

**Published:** 2013-09-15

**Authors:** Li-Qun Wang, Zhao-Sheng Chen

**Affiliations:** School of Energy and Power Engineering, Jiangsu University, 301 Xuefu Road, Zhenjiang 212013, China

## Abstract

This paper presents the experimental results of cogasification of coal and biomass in an intermittent fluidized bed reactor, aiming to investigate the influences of operation parameters such as gasification temperature (T), steam to biomass mass ratio (SBMR), and biomass to coal mass ratio (BCMR) on hydrogen-rich (H_2_-rich) gas production. The results show that H_2_-rich gas free of N_2_ dilution is produced and the H_2_ yield is in the range of 18.25~68.13 g/kg. The increases of T, SBMR, and BCMR are all favorable for promoting the H_2_ production. Higher temperature contributes to higher CO and H_2_ contents, as well as H_2_ yield. The BCMR has a weak influence on gas composition, but the yield and content of H_2_ increase with BCMR, reaching a peak at the BCMR of 4. The H_2_ content and yield in the product gas increase with SBMR, whilst the content of CO increases first and then decreases correspondingly. At a typical case, the relative linear sensitivity coefficients of H_2_ production efficiency to T, SBMR, and BCMR were calculated. The results reveal that the order of the influence of the operation parameters on H_2_ production efficiency is T > SBMR > BCMR.

## 1. Introduction

Hydrogen is likely to be an important energy carrier in the future [1]. However, the presence of hydrogen on Earth is limited to hydrogen-containing compounds and today, nearly 96% of hydrogen is produced from fossil fuels, which emit a large amount of carbon dioxide [2–4]. Conversely, biomass represents a clean and renewable energy resource with a carbon dioxide neutral effect on the environment. Therefore, biomass has been known as the most potential material for hydrogen production in the future [5].

Biomass gasification process has emerged as a clean and efficient way of producing hydrogen [6]. It is a well-known technology that can be classified depending on the reactor: fixed bed, moveable bed, fluidized bed, and so forth. The fluidized bed reactor has been widely used as the biomass gasifier to produce hydrogen due to its various advantages which include better temperature control, wide feedstock adaptability, good gas solids contact, and excellent heat and mass transfer characteristics. Different gases such as air [7–9], pure steam [10–12], and air-steam [13, 14] can be adopted as the gasifying mediums.

The addition of steam as a gasifying medium in gasification process makes it possible to obtain high-grade and nearly N_2_-free H_2_-rich gas [15]. However, there are a number of potential problems [16, 17] in biomass gasification with steam [16] as follows. (i) If the biomass reacts with both steam and air in one reactor, then nitrogen is present in product gases and is costly to remove [13, 14]. (ii) If one tries to avoid this problem by using pure oxygen instead of air, then a source of pure oxygen would be needed, which is again a costly option [8]. (iii) It is possible to circumvent the separation “issues” by running “oxygenless” gasification and the combustion reactions in different locations, but then transferring heat from one location to the other is accompanied with heat losses [18–21]. In order to meet the heat needs of gasification, the combustion reactor requires higher temperature to compensate the heat losses [22].

In order to overcome the problems, a process concept which involves time-segregated hybridization of combustion/gasification reactions in one fluidized bed reactor is proposed. This process consists of two separate stages, that is, combustion and gasification, in a reactor. Specifically, the combustion stage is used for coal combustion fed with air. The biomass and pure steam are added in the gasification stage to serve as the gasification raw material during gasification. Direct contact between the combustion and gasification stages is avoided. This gasification technology operates in the same reactor; thus all the energy of coal combustion can be supplied in to the gasification stage, which proves to be better for tar cracking, and also can effectively prevent sintering of the raw materials in gasification process.

The objective of this study was to investigate the influences of T, SBMR, and BCMR on H_2_-rich gas production by cogasification of coal and biomass in an intermittent fluidized bed. Additionally, a sensitivity study was employed in order to provide a comparable measure of the influence of T, SBMR, and BCMR on H_2_ production efficiency.

## 2. Experimental Part

### 2.1. Raw Materials

In this study, corn core and lean coal obtained from Jiangsu Province, China, were employed as biomass and coal feedstock, respectively. The mean diameter of the biomass particles is 5 mm, whereas that of the coal particles is 3 mm. The proximate and ultimate analyses of the raw materials are reported in Table 1.

### 2.2. Facilities

**Figure 1:**
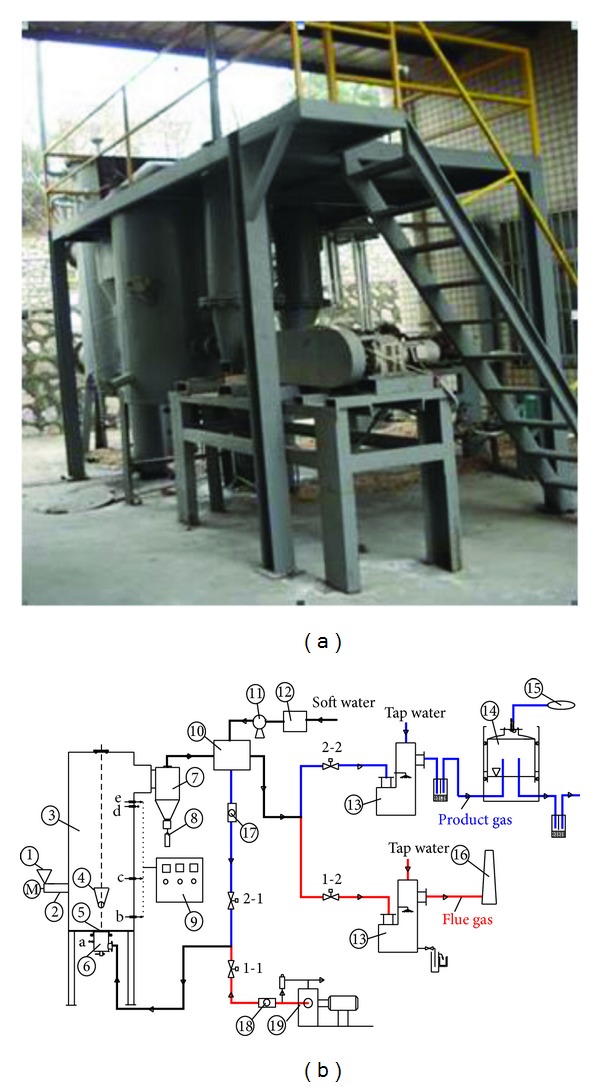
Cogasification of coal and biomass in an intermittent fluidized bed reactor. (a) Pilot plant, (b) schematic diagram: (1) biomass hopper; (2) screw feeder; (3) fluidized bed reactor; (4) coal feeder; (5) air distributor; (6) wind room; (7) high-temperature cyclone separator; (8) char collector; (9) PID temperature controller; (10) tube-type heat exchanger; (11) soft water pump; (12) soft water tank; (13) wet scrubber; (14) bell-type gas holder; (15) product gas collection bag; (16) chimney; (17) steam flow meter; (18) air flow meter; (19) roots blower, (b), (c) and (e): k-type thermocouple, (a) and (d): pressure taps; 1-1: air control valve (solenoid valve); 1-2: flue control valve (pneumatic valve); 2-1: steam control valve (solenoid valve); 2-2: gas control valve (pneumatic valve).

The experimental system, whose schematic diagram is illustrated in Figure 1, consists of five main parts: (a) a fluidized bed reactor, (b) a temperature control section, (c) two feeding sections, (d) air and steam supplying sections, and (e) gas purifying, sampling, and metering sections. The fluidized bed reactor is a cylindrical, stainless steel shell with a supporting structure. The total height of the fluidized bed is 2000 mm and the inner diameter (id) is 300 mm. Two pressure taps are mounted at the center of wind room and at the height of 1006 mm above the air distributor to monitor the pressure drop of the fluidized bed reactor. The bed temperatures at three different points along the height of 160, 600, and 1056 mm above the distributor are measured by K-type thermocouples with a diameter of 18 mm. At the bottom of the fluidized bed reactor, an air distributor is installed for better air distribution. The distributor is 16 mm in thickness with 126 holes (id = 2 mm) perforated uniformly on it. The coal and biomass are fed separately to the fluidized bed reactor by two different screw feeders. The feeding point is at 480 mm above the air distributor. The feeder pipes have external water-cooling heat exchanger to avoid raw materials pyrolysis before they enter the fluidized bed reactor. Air is introduced into the fluidized bed reactor below the distributor, as an oxidizing medium for coal combustion; in combustion stage, it is provided by a roots blower. The steam of 300°C is used as the gasification medium and also introduced from the bottom but in gasification stage, it is provided by a steam generator, and its mass flow rate is measured by a steam flow meter. The solid particulates (ash, dust, and char) from the high-temperature hot gas are separated by the high-temperature cyclone separator and collected at the bottom. The high-temperature hot gas is cooled to about 200°C by a water-cooled shell and tube-type heat exchanger. The gas then passed through a wet scrubber to condense organic vapors (tar) and further cool gas with tap water. Finally, the purified gas is introduced into the bell-type gas holder to storage.

### 2.3. Procedures

The technology divides the production process into combustion and gasification stages in the same reactor. Through two pairs of control valves (see Figure 1), namely, 1-1 and 1-2 valves and 2-1 and 2-2 valves, two working stages are formed. During combustion stage, the first pair of valves is opened. Coal and air are added to the reactor when coal is combusted in fluid state, and the reactor temperature (or bed temperature) rapidly increases. When the bed temperature rises to the desired temperature and remains steady, the combustion stage ends, the air and coal supply ceases, and the first pair of valves is closed. Meanwhile the second pair of valves is successively opened. Biomass and steam are introduced to the reactor, thus inducing the high-temperature materials to undergo pyrolysis gasification reaction with pure steam in a fluid state. This produces H_2_-rich gas. Given that the reaction is of an intensive endothermic nature, the bed temperature rapidly declines. When the bed temperature decreases to the predetermined value, the gasification stage ends, the biomass and steam supply ceases, and the second pair of valves is closed. The control valves then return again to the combustion stage. This cycle is then repeated.

Both stages occur by controlling the two pairs of control valves dominated by the preprogrammed temperatures. 

In gasification stage, the sample of the product gas was collected from the bell-type gas holder with gas bag and analyzed for major components (H_2_, CO, CO_2_, and CH_4_) by gas chromatograph (GC-2010, Shimadzu International Trading (Shanghai) Co, Ltd, China).

## 3. Results and Discussion

### 3.1. Influences of Temperature

The reaction temperature is an important factor with regard to the final composition of the gases and products distribution [23]. The main reactions in coal and biomass cogasification are listed as follows [24]:

(R1) Oxidation:Coal+Air   →combustionHeat+Ccoal+CO+CO2+N2+⋯

(R2) Pyrolysis:Biomass+Heat(from  R1) →pyrolysisCbiomass+Tar+Gases

(R3)  Water-gas:(Ccoal+Cbiomass)+H2O⇔CO+H2

(R4)  Boudouard:(Ccoal+Cbiomass)+CO2⇔2CO

(R5)  Water-gas  shift:CO+H2O⇔CO2+H2

(R6)  Steam  reforming:CH4+H2O⇔CO+3H2

(R7)  Tar+H2O→decompositionGases(H2,CO,CO2,CH4,CnHm)

(R8)  Methanation:(Ccoal+Cbiomass)+2H2⇔CH4

**Figure 2:**
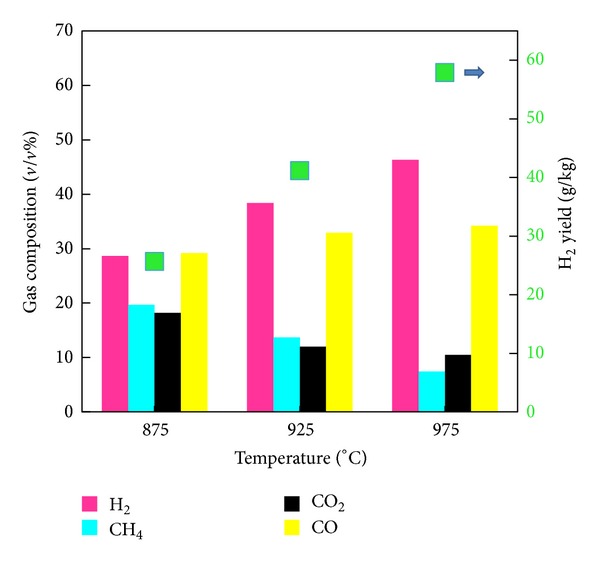
Influence of temperature on H_2_-rich gas production.

Figure 2 shows the influences of temperature on H_2_-rich gas production at SBMR of 1.3 and BCMR of 4. It is found that the H_2_ content and yield in the product gas increased from 28.7% and 25.63 g/kg at 875°C to 46.3% and 57.88 g/kg at 975°C, respectively. The main reason for the increase in H_2_ output is that the water gas reaction ((R3)) is promoted with increasing temperature.

Under steam coal and biomass cogasification conditions, the endothermic char gasification with water reaction ((R3)), the Boudouard char gasification with CO_2_ reaction ((R4)), and CH_4_ steam reforming reaction ((R6)) are accelerated with an increase in temperature resulting in an increase of CO and H_2_ and a decrease in CH_4_ and CO_2_. Moreover, H_2_ is greater than that of CO, and the difference between the two gas contents increased with the rise of temperature. The presence of steam favors water gas shift reaction ((R5)) leading to an increase in H_2_ content and a decrease in CO concentration with the increase of temperature. Although ((R6)) reaction also releases CO_2_, the CO_2_ concentration decreases with the increase of temperature; this may be attributed to the ((R4)) reaction that consumes CO_2_ in return, resulting in an increase of CO concentration and a decrease in CO_2_ concentration with increased temperatures [25]. The experimental results seem to show that the ((R6)) reaction is less important for higher temperature range; thus the ((R3)) and ((R4)) reactions have a more prevailing role. 

Higher temperature contributes to higher H_2_ content and yield. It seems to indicate that the temperature should be kept at the highest possible value. However, we should keep in mind that higher temperature would result in other problems, such as higher operational costs and high temperature agglomeration. Therefore, proper high temperature should be considered for cogasification of coal and biomass in fluidized bed reactor.

### 3.2. Influences of SBMR

**Figure 3:**
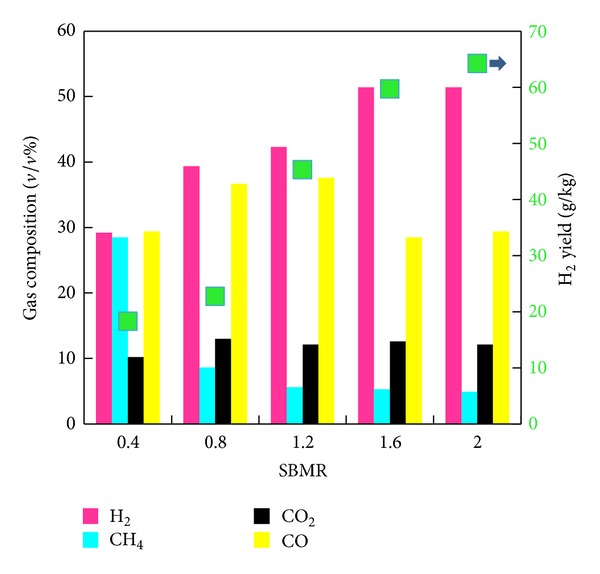
Influence of SBMR on H_2_-rich gas production.

Figure 3 presents the influences of SBMR on H_2_-rich gas production at BCMR of 4 and 950–1000°C. With the increase of SBMR from 0.4 to 2, the H_2_ content and yield in the product gas increased from 29.2% and 18.25 g/kg to 51.4% and 64.25 g/kg, respectively. Contrary to the results obtained here, Li et al. [26] found a decrease in H_2_ yield when SBMR increased from 0.2 to 1. They contributed their observation to the decreased temperature resulting from heat absorbing by the excess water in their fluidized bed reactor [4]. However, for each test in this study, the temperature inside the fluidized bed reactor kept constant by taking advantage of the coal combustion, ensuring the influences of SBMR on H_2_-rich gas production avoiding the interference of varied temperature. The water gas reaction ((R3)) and all steam reforming reactions are strengthened with an increase of SBMR, which resulted in increase in H_2_ yield.

In regard to the gas composition, with SBMR varying from 0.4 to 2, H_2_ content increased from 29.2% to 51.4%, CH_4_ decreased from 28.5% to 4.9%, while CO_2_ varied little. In particular, the variation of CO content did not show a monotonic trend. Molar fraction of CO rose as SBMR increased from 0 to 1.2 but declined as SBMR went up. Thus, maximum carbon monoxide content reached 37.6% as SBMR is 1.2. This result is related to the fact that the contents of the H_2_, CO, and CO_2_ were linked together by the equilibrium of the ((R5)) reaction in the current test conditions.

With regard to the changing trend of gas components with the varied SBMR in coal and biomass cogasification, some researchers reported different phenomena [27, 28]. The difference possibly came from the different reactor, raw materials, operating conditions, and gasifying agents used.

### 3.3. Influences of BCMR

**Figure 4:**
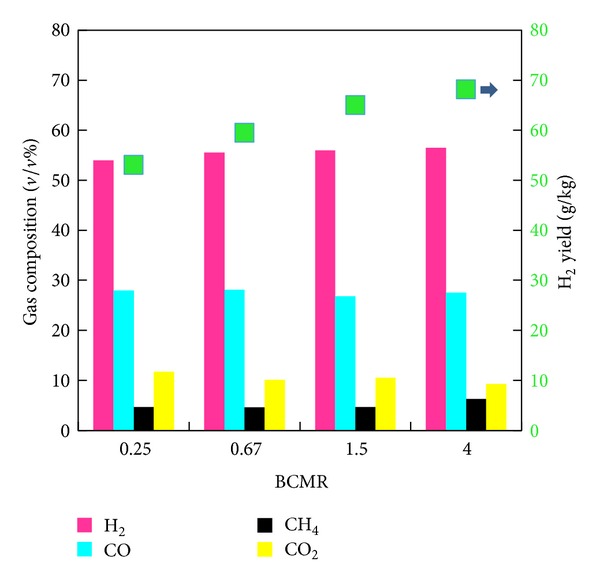
Influence of BCMR on H_2_-rich gas production.

Figure 4 gives the influences of BCMR on H_2_-rich gas production. It is found that the H_2_ content and yield in the product gas increased from 54% and 53.04 g/kg at 0.25 to 56.5% and 68.13 g/kg at 4, respectively. Maximum H_2_ content reached 56.5% as BCMR is 4. The result indicates that the total amount of the residual char after the combustion stage together with the biomass char makes water gas reaction dominate the gasification stage, which generates the highest amount of H_2_ when BCMR = 4. The other gas species like carbon monoxide (28%–27.5%), carbon dioxide (4.7%–6.3%), and carbon dioxide (11.7%–9.3%) are independent of the BCMR.

## 4. Sensitivity Analysis

Three operation parameters mentioned in Sections 3.1, 3.2, and 3.3 have different degree influences on hydrogen production. The hydrogen production efficiency (*η*_H_2__) defined as (1) is used to characterize the quality of hydrogen production. The relative linear sensibility coefficient defined as (2) is used to evaluate the influences of these operating parameters on H_2_ production process [29]:

(1) $$\begin{array}{c} \eta_{{\text{H}}_{2}}=\frac{m_{{\text{H}}_{2}}\cdot {\text{LHV}}_{{\text{H}}_{2}}}{m_{F}\cdot {\text{LHV}}_{F}}, \end{array}$$

where *m*_*F*_ and *m*_H_2__ represent the mass of fuel and H_2_, respectively. LHV_*F*_ and LHV_H_2__ are the lowest heating values of fuel and H_2_, respectively,

(2) $$\begin{array}{c} \omega_{\text{i}}=\frac{\Delta \eta_{{\text{H}}_{2}}/\eta_{{\text{H}}_{2}}}{\Delta X_{i}/X_{i}}, \end{array}$$

where *X*_*i*_ is the operating parameter of number *i*.

**Figure 5:**
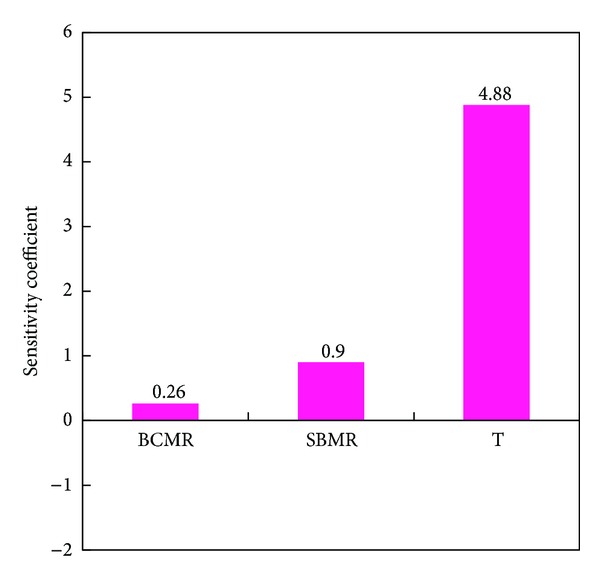
Relative linear sensitivity coefficients of H_2_ production efficiency towards the operation parameters.

The relative linear sensitivity coefficients of H_2_ production efficiency to the operation parameters such as T, SBMR, and BCMR were calculated which are given in Figure 5. As presented in Figure 5, the order of the influence of the operation parameters on H_2_ production efficiency is T > SBMR > BCMR. It is found that the sensitivity coefficients of T, SBMR, and BCMR are positive.

Consequently, the control of hydrogen production process should start from controlling T. Moreover, the increase of SBMR is helpful for the H_2_ production.

## 5. Conclusions

The aim of this work was to investigate the influences of T, SBMR, and BCMR on H_2_-rich gas production by cogasification of coal and biomass in an intermittent fluidized bed. Additionally, a sensitivity study was employed in order to provide a comparable measure of the influence of T, SBMR, and BCMR on H_2_ production efficiency. The remarkable conclusions are ordered as follows.

- This technology exhibits the characteristics of stability and reliability in the industrialization process. Over the ranges examined in this study, the increases of T, SBMR, and BCMR were all favorable for promoting the H_2_ production.
- The H_2_ and the CO contents increase with increasing temperature, whilst the contents of CO_2_ and CH_4_ decrease correspondingly.
- With increasing SBMR, the H_2_ content and yield in the product gas increase. CH_4_ in the gas decreases with SBMR, whilst CO increases first and then decreases correspondingly.
- The BCMR has a weak influence on composition of gas produced, but the yield and content of hydrogen increase with increasing BCMR, reaching a peak at the BCMR of 4.
- The order of the influence of the operation parameters on H_2_ production efficiency is T > SBMR > BCMR.

## Figures and Tables

**Table 1:** Proximate and ultimate analyses of biomass and coal.

Samples	Proximate analysis/wt.%				Ultimate analysis/wt.%			
	Vol.	Mois.	FC	Ash	C	H	O	N
Lean coal	5.5	4.7	41.86	25.3	62.42	2.83	1.9	1.02
Corn core	67.8	5.6	9.9	5.9	47.53	4.9	37.75	0.85
